# Predictors of time until return to work and duration of sickness absence in sick-listed precarious workers with common mental disorders: a secondary data-analysis of two trials and one cohort study

**DOI:** 10.1186/s13033-023-00613-7

**Published:** 2023-12-08

**Authors:** Yvonne B. Suijkerbuijk, Frederieke G. Schaafsma, Lyanne P. Jansen, Selwin S. Audhoe, Lieke Lammerts, Johannes R. Anema, Karen Nieuwenhuijsen

**Affiliations:** 1 Department of Public and Occupational Health, Amsterdam Public Health Research Institute, Amsterdam UMC, location University of Amsterdam, Meibergdreef 9, Amsterdam, 1105 AZ The Netherlands; 2 Research Center for Insurance Medicine, AMC-UMCG-VUmc-UWV, Amsterdam, The Netherlands; 3 Department of Public and Occupational Health, Amsterdam Public Health Research Institute, Amsterdam UMC, location Vrije Universiteit Amsterdam, Van der Boechorststraat 7, Amsterdam, 1081 BT The Netherlands

**Keywords:** Mental Health, Vocational Rehabilitation, Return to work, Sick leave, Social Security

## Abstract

**Background:**

Common mental disorders (CMD) are highly prevalent among sick-listed precarious workers and often lead to long-term sickness-absence, work disability and unemployment. This study aimed to identify predictors of a longer time until return to work (RTW) and prolonged duration of sickness absence in sick-listed precarious workers with CMD.

**Methods:**

We conducted a secondary Cox regression analysis using existing data from two Dutch randomized controlled trials and one cohort study among sick-listed precarious workers with CMD (N = 681). Age, gender, baseline employment status, study allocation, severity of psychological symptoms and RTW self-efficacy were evaluated for their predictive value on time until sustainable (≥ 28 days) RTW and duration of sickness absence during 12-month follow-up. In this study, time until sustainable RTW and duration of sickness absence are distinct dependent variables, because they are not mutually exclusive.

**Results:**

Age above 50 years (HR 0.57, 95% CI 0.39–0.82), severe psychological symptoms (HR 0.64, 95% CI 0.43–0.93), unemployment (HR 0.19 95% CI 0.11–0.33) and loss of employment contract during sickness absence (HR 0.25, 95% CI 0.14–0.47) were predictive of a longer time until RTW. Male gender (HR 0.77, 95% CI 0.62–0.97), severe psychological symptoms (HR 0.64, 95% CI 0.46–0.87), unemployment (HR 0.47, 95% CI 0.27–0.84) and loss of employment contract (HR 0.48, 95% CI 0.26–0.90) predicted a prolonged duration of sickness absence.

**Conclusions:**

Unemployment at the moment of sick-listing, loss of employment contract during sickness absence, and severe psychological symptoms are predictors of both a longer time until RTW and prolonged duration of sickness absence among sick-listed precarious workers with CMD. This knowledge assists occupational health and mental health professionals in the early identification of workers at risk of long-term sickness absence, enabling them to arrange targeted occupational rehabilitation support and mental health care.

**Trial registration:**

The included randomized controlled trials were prospectively registered in the Dutch national trial register under NTR4190 (September 27, 2013) and NTR3563 (August 7, 2012).

**Supplementary Information:**

The online version contains supplementary material available at 10.1186/s13033-023-00613-7.

## Background

Mental disorders are highly prevalent in the general and working-age population [1, 2]. While definitions of common mental disorders (CMDs) differ from one study to another, the population life-time prevalence of CMDs, including depression, anxiety, stress-related disorders and substance abuse, is as high as 30% [2, 3]. Mental disorders often lead to recurring or long-term sickness absence, work disability and unemployment [4]. However, most people with a mental disorder do wish to engage in work [5, 6]. Moreover, it is well known that employment contributes to mental health and wellbeing: in addition to financial security, employment provides daily structure, social contacts, de-stigmatization, and also enhances mental health, general wellbeing, self-esteem and sense of identity [7–9], while remaining unemployed can contribute to financial problems and social isolation, possibly leading to further decrease of mental health [10]. Thus, to enhance the mental and social wellbeing of workers with mental disorders and to diminish working disability costs, it is important that these workers receive adequate occupational health care to support work participation [4, 8].

In high-income countries, sick-listed workers with a permanent employment contract often receive occupational health care to support successful return to work (RTW). Contrarily, for sick-listed workers without a permanent contract, access to occupational health care is limited [11]. This is concerning, because the prevalence of non-permanent work, often referred to as precarious employment, is increasing due to globalization and the flexibilization of the labor market [11, 12]. Precarious employment is generally defined as work that is uncertain, unstable and insecure, while workers bear work-related risks and receive limited social benefits and statutory protection [13]. Examples of precarious employment are temporary work or own-account self-employment. Compared to permanently employed workers, precarious workers generally have a lower socioeconomic position, encounter more psychosocial barriers and experience worse health [11, 14, 15]. As a result, return to (new) work is more challenging for this vulnerable group of workers [16].

To facilitate RTW and prevent long-term sickness absence, it is important to obtain more knowledge about barriers to RTW for precarious workers with mental disorders, so that targeted occupational rehabilitation interventions can be developed. Predictive factors of a longer time until RTW and prolonged duration of sickness absence can be regarded as risk factors for long-term sickness absence, but may also give insight into potential facilitators of and barriers to RTW. In the last few decades, several predictive factors of RTW in employed workers with mental disorders have been identified. These factors can be divided into health-related, work-related and personal factors [17–19]. Most of these studies were solely conducted among permanently employed workers. Unfortunately, only two previous cohort studies evaluated predictors of RTW and sickness absence among sick-listed precarious workers [20, 21]. RTW-expectations were found to be predictive of RTW and long-term sickness absence in both studies. Other predictors were age and perceived health, in the study of Audhoe et al. [20], and educational level and help-seeking behavior, in the study of Louwerse et al. [21]. Evidence concerning effective interventions to enhance RTW and prevent working disability for sick-listed precarious workers with CMD is also limited [22–24].

The current study aimed to identify predictors of a longer time until RTW and prolonged duration of sickness absence among Dutch sick-listed precarious workers with CMD. We evaluated the following potential predictors: age, gender, previous employment status, occupational rehabilitation intervention, severity of psychological symptoms and RTW self-efficacy.

## Methods

### Design

To identify predictors of a longer time until RTW and prolonged duration of sickness absence, we conducted secondary analyses based on the existing data of two independent Dutch randomized controlled trials (RCTs) [23, 24] and one Dutch cohort study [Lammerts et al., unpublished] carried out between 2013 and 2017. These previous studies evaluated RTW and sickness absence in sick-listed precarious workers with CMD. Data about demographics, health and work were also collected. In the current study we combined these data in a secondary data-analysis to identify which factors are predictive of a longer time until RTW and prolonged duration of sickness absence. The primary dependent variable was days until RTW during 12-month follow-up. The secondary dependent variable was days until end of sickness absence during 12-month follow-up. The primary studies were approved by the Medical Ethics Committee of Amsterdam UMC and all participants signed informed consent for future use of their data for research. The two RCTs were registered in the Dutch national trial register under NTR4190 (Brainwork study, Audhoe et al.) and NTR3563 (Co-WORK, Lammerts et al.). We used the TRIPOD-checklist (Transparent Reporting of a multivariable prediction model for Individual Prognosis Or Diagnosis) as a reporting guideline [25].

### Study population

All participants of the three included studies were precarious workers with CMD, who were newly sick-listed at The Dutch Social Security Institute: the Institute for Employee Benefits Schemes (UWV). In the Netherlands, the UWV provides sickness benefits and occupational health care for precarious workers who are not (fully) able to work due to illness. In the current study, we define precarious employment as non-permanent work such as temporary agency work, self-employment and loss of employment contract before or during sickness absence. We define CMDs as mild to moderate mental disorders that occur frequently among adults, such as depression, anxiety and adjustment disorders.

The participants of the RCT (N = 186) of Lammerts et al. (Co-WORK study) [23] were sick-listed precarious workers with CMDs, with an intention to return to work despite ongoing health complaints. Sick-listed precarious workers with CMD without intention to return to work were followed up in a cohort. The data of this cohort study (N = 177) were also included in the secondary analyses of the current study. In the RCT of Audhoe et al. (Brainwork study) [24] sick-listed precarious workers with CMDs who did not expect to return to work within two weeks (N = 320) were included. Audhoe et al. excluded workers with a severe mental disorder or substance abuse disorder as main reason for sickness absence. Lammerts et al. excluded in both studies workers with an ongoing conflict with the UWV or a legal conflict. All three studies excluded workers with pregnancy up to three months after delivery.

The participants of these three studies were recruited through several UWV offices across the Netherlands. In the Brainwork study, local insurance physicians evaluated the eligibility of the participants for inclusion in the study. The eligibility check in the Co-WORK and cohort study was performed by the researchers, by evaluating a self-report questionnaire. The participants in the intervention groups of the two RCTs were allocated to a RTW-intervention and the participants in the control group received occupational health care as usual The RTW intervention in the CO-WORK study included a participatory approach, integrated care and direct placement in a paid job [23]. The Brainwork Intervention was characterized by an activating approach, protocol-based stepped care and intensive vocational counseling [24].

### Data collection

The data of the three previous studies were retrieved based on a data-sharing agreement. The data were then merged and used for the secondary data analyses. For the purpose of pseudonymization all participants were recoded using random numbers. Additionally, we transformed time dates into continuous data (for example age instead of date of birth).

All participants of the three previous studies with available data about ≥ 1 dependent variable(s) and ≥ 1 independent variable(s) (N = 681/683) were included in the analyses. Data sources included both registered data from the computerized database of the UWV and paper-based self-reported questionnaires (see Table 1).

**Table 1:** Data sources

Variable	Registered data	Self-reported questionnaires
Age	X (Brainwork)	X
Gender	X (Brainwork)	X
Employment status	X	
Education level^a^		X
Psychological symptoms		X
RTW self-efficacy		X
RTW expectations^a^		X
Participation level	X	
Time until sustainable RTW	X	
Duration of sickness absence^b^	X	X (cohort)

^a^Education level and RTW expectations were not included in the regression analyses, because data were not available for the participants of the Brainwork study (Audhoe et al.) [24]

^b^Based on first until last day of being sick-listed. The Co-WORK study [23] initially used the number of days between study enrolment until ending of the sickness benefit for ≥ 28 days

### Dependent variables (outcomes)

In the current study, time until RTW and duration of sickness absence are distinct dependent variables, because these variables are not mutually exclusive. Under the Dutch social security system, workers can leave sickness absence, even though they have not yet (fully) returned to work or still perceive some symptoms. Generally, the sickness absence period ends if a worker considers him- or herself to be recovered or if an insurance physician declares the worker fit for work. Furthermore, Dutch precarious workers cannot be on partial sick leave, they are either on full sick leave or not at all.

We defined (sustainable) RTW as being returned to work or re-employed of at least 28 consecutive days, This definition was also used in the two included studies of Lammerts et al. based on the Dutch Sickness Benefit Act. This time frame of ≥ 28 days is also the most common used definition of sustainable RTW in Northern European studies [26, 27]. Data about time until sustainable RTW were obtained from registered data of the UWV. The follow-up period was 365 days, starting on the first day of being sick-listed. We calculated the time until sustainable RTW based on the number of days from the day of being sick-listed to the day of returning to full-time or part-time competitive work for ≥ 28 consecutive days.

The definition and data sources regarding the duration of sickness absence differed somewhat between the three studies (see Table 1). We defined and recalculated the duration of sickness absence as the number of days from the first day of being sick-listed up to and including the last day of being sick-listed during follow-up.

### Independent variables (potential predictors)

Baseline employment status was based on registered data of the UWV and includes three different states: unemployment at the time of sick-listing, loss of employment contract during sickness absence and temporary agency work. Data regarding age and gender were based on registered data of the Brainwork study and self-report data of the Co-WORK and cohort study. For age we differentiated between young to middle aged workers (< 50 years) and older workers (≥ 50 years) in the regression analyses. For gender solely binary data (male/female) were available. Type of study and study allocation (intervention, control, cohort) were also included as potential predictors. Psychological symptoms and RTW self-efficacy were derived from self-reported questionnaires and the sum scores were dichotomized to differentiate between high and low scores.

#### Psychological symptoms

For severity of psychological symptoms Audhoe et al. [24] used the Dutch translation of the General Health Questionnaire-12 (GHQ-12) [28] and Lammerts et al. [23] used the Four-Dimensional Symptom Questionnaire (4DSQ) for both studies [29]. These self-report instruments are commonly used and validated for the screening of mental health symptoms in community care. The GHQ-12 has shown acceptable validity and reliability as a unidimensional index of severity of common mental disorders [28, 30]. The 4DSQ has been validated in Dutch primary care patients with psychological distress, compromising four scales of symptoms (distress, depression, anxiety and somatization) [29, 31].

In the current study, we dichotomized the severity of psychological symptoms into mild-to-moderate symptoms and severe symptoms using the median sum score of the GHQ12 as cut-off point. Of the 4DSQ data we used the existing scoring system of Terluin et al. [29, 32] to differentiate between mild-to-moderate symptoms and severe symptoms. A sum score of > 6 on the depression scale and/or > 12 on the anxiety scale was considered as severe symptoms.

#### RTW self-efficacy

Self-efficacy is a judgement regarding one’s own ability to succeed in a specific behavior [33]. Applied to RTW, self-efficacy can be explained as the belief that workers have in their own ability to meet the demands made by a RTW [34]. Audhoe et al. [24] used the 11-item RTW-SE scale [234. The RTW-SE scale shows good psychometric properties regarding reliability and construct validity [34, 35]. Lammerts et al. [23] used a (non-validated) questionnaire developed by van Oostrom and colleagues [36] based on the Attitude-Social influence-self-Efficacy (ASE)-model [37] including questions about attitude, normative beliefs, social modelling, self-efficacy and fear avoidance beliefs. For the secondary analyses we dichotomized the scores to create two groups: those with a low level of self-efficacy and those with a high level of self-efficacy at baseline, based on the sum scores of the 11-item RTW-SE scale and the self-efficacy scale of the ASE questionnaire. The median scores were used as cut-off point to differentiate between a low and high level of RTW self-efficacy.

### Statistical analysis

Descriptive statistics are presented by means (SD) for normally distributed numerical data, medians (IQR) for not-normally distributed numerical data and numbers (percentages) for categorical data. We evaluated normality of data graphically with histograms and statistically with Skewness and Kurtosis tests.

To determine the association between the baseline characteristics and the (potentially censored) time-to-event data, we used Kaplan Meier and Cox regression analyses. Baseline characteristics measured in all three studies and available for > 50% of the participants were included as independent variables in the univariable analyses to test their associations with the dependent variables. Unadjusted Kaplan Meier survival curves were plotted to visualize the time until event for each independent variable. The time until 50% of the participants reached an event (median survival time) was calculated. Where < 50% of the participants reached an event, we assessed the duration until the event was reached by ≥ 25% (25th percentile) of the participants. We used Cox regression analyses to evaluate the influence of the independent variables on days until sustainable RTW and days until end of sickness absence. Hazard ratios (HRs), with their 95% confidence intervals and *p*-values, show the division of the probability of an event for a group of participants compared to the participants in the reference category at each time point during 12-month follow-up. The reference category is the category with the highest event rate during follow-up. An HR < 1 indicates a lower event rate at each time point during follow-up, and thus a longer time until event during follow-up compared to the reference category. We also calculated the probabilistic index (PI) based on the following formula: PI = 1/(1 + HR). The PI indicates the probability of a longer time until RTW compared to the reference group [38].

Independent variables with *p* < 0.2 in the univariable Cox regression analyses were included in the multivariable Cox regression analysis. A cut-off value of *p* < 0.05 (Wald statistic) was used to determine the significance of the associations in the multivariable analysis. The included independent variables in the multivariable analysis were tested for multicollinearity using variance inflation factors (VIF), considering a VIF-score < 10 as acceptable [39]. We evaluated the assumption of proportional hazards graphically by creating log-minus-log survival plots. Furthermore, we assessed the proportionality by using partial Schoenfeld residuals and inclusion of time-varying covariates. All statistics were performed in IBM SPSS Statistics for Windows, Version 26.0.

### Missing value analysis

For variables with ≥ 5% missing values, we evaluated whether the nature of the missing data was completely at random (MCAR), ad random (MAR) or not at random (NMAR) [40, 41]. For this, we inspected the pattern and distribution of the missing data and we searched for differences between participants with and without missing data (based on t-tests and Pearson Chi ²-tests). In addition, we performed the Little’s MCAR test [42] to check if the data were missing completely at random. Lastly, logistic regression analyses were performed to test whether the missing data could be predicted and thus be considered as MAR. This was based on the Nagelkerke R² statistic [43].

Multiple imputation by chained equations (MICE) [44] was used because the missing data were likely to be MAR, the number of the missing data exceeded 5% for two variables and the patterns did not show monotonicity. The number of imputation sets was similar to the percentage of missing values (rule of thumb) [44]. The pooled results based on the Wald statistic were used for the variable selection [41, 45]. Participants with no RTW or end of sickness absence within 12-month follow-up were regarded as censored and received a value of 365 days for time until RTW or duration of sickness absence.

## Results

### Descriptive statistics

In total 681 participants of the three previous studies were included in the secondary analyses. Most participants were already unemployed before being sick-listed (81%) and almost half of the participants experienced severe psychological symptoms. Table 2 shows all baseline characteristics of all participants. For the original scores of the psychological symptoms (GHQ12 and 4DSQ) and RTW self-efficacy (RTW-SE, ASE questionnaire) see Additional file l.

**Table 2:** Baseline characteristics (N = 681)

Baseline characteristics	Total (N = 681) n (%)	Brainwork (N = 320) n (%)	Co-WORK (N = 186) N (%)	Cohort (N = 175) (n (%)
*Age*				
<50 years Mean years (SD)	458 (67) 44 (11)	256 (80) 41 (11)	103 (55) 46 (10)	99 (57) 47 (10)
*Gender*				
Female Male	351 (52) 330 (48)	173 (54) 147 (46)	92 (49) 94 (51)	86 (49) 89 (51)
*Employment status (baseline)*				
Unemployed Temporary Agency contract Employed, loss of contract during sickness absence	553 (81) 19 (3) 109 (16)	216 (68) 11 (3) 93 (29)	173 (93) 6 (3) 7 (4)	164 (94) 2 (1) 9 (5)
*Psychological symptoms* ^a^				
Mild-to-moderate Severe Unknown	131 (19) 319 (47) 231 (34)	39 (12) 50 (16) 231 (72)	51 (27) 135 (73) -	41 (23) 134 (77) -
*RTW self-efficacy* ^b^				
High Low Unknown	170 (25) 276 (41) 235 (34)	40 (13) 45 (14) 235 (73)	105 (57) 81 (43) -	25 (14) 150 (86) -
*Type of study*				
Brainwork Co-Work Cohort	320 (47) 186 (27) 175 (26)	320 (100) - -	- 186 (100) -	- - 175 (100)
*Study allocation*				
Intervention Control Cohort	258 (38) 248 (36) 175 (26)	164 (51) 156 (49) -	94 (51) 92 (49) -	- - 175 (100)
*Expected RTW < 6 months* ^c^ *(N = 361)*	85 (24)	-	20 (11)	65 (37)
*Education (low)* ^c^ *(N = 361)*	104 (29)	-	49 (26)	55 (31)

^a^ Cut-off point based on the median of the GHQ12 (Brainwork participants) and 4DSQ (Cohort and Co-Work participants)

^b^ Cut-off point based on the median of the RTW-SE scale (Brainwork participants) and ASE-SE subscale (Cohort and Co-Work participants)

^c^ Expected RTW < 6 months and level of education were not included in the analyses because data were not available for any of the participants from the Brainwork study. Low education level included no education, primary school or lower vocational education

The work participation level of the participants during 12-month follow-up is presented in Table 3. During 12-month follow-up, 179 participants (26%) had a sustainable RTW (≥ 28 consecutive days). For the participants who returned to work sustainably, the median time until RTW was 185 (IQR 94–262) days. About 64% of the participants without sustainable RTW and 13% with sustainable RTW were still sick-listed after 12 months. Thus, leaving sickness absence does not always imply sustainable RTW, and vice versa. For the participants who were no longer sick-listed after 12 months, the median duration of sickness absence was 144 (IQR 82–240) days. The univariable (unadjusted) survival curves regarding time until sustainable RTW and duration of sickness absence are presented in Additional file 2 and 3.

**Table 3:** Work participation and sickness absence during 12-month follow-up (N = 681)

Variables	n	%	median	IQR
*Highest level of participation (during follow-up)*				
Sustainable RTW Non-sustainable RTW Return to non-competitive work No participation	179 18 32 452	26 3 5 66		
*Median days until sustainable RTW*				
All participation levels Sustainable RTW	681 179	100 26	365^b^ 185	335–365 94–262
*Duration of sickness absence*				
<12 months ≥ 12 months^a^ Unknown	314 343 24	46 50 4		
*Duration of sickness absence in participants with sustainable RTW (N = 179)*				
<12 months sickness absence ≥ 12 months sickness absence^a^ Unknown	145 24 10	81 13 6		
*Duration of sickness absence in participants without sustainable RTW (N = 502)*				
<12 months sickness absence ≥ 12 months sickness absence^a^ Unknown	169 319 14	34 64 3		
*Median days of sickness absence period*				
All participants (with available sickness absence data) Participants with < 12 months sickness absence Participants with sustainable RTW (with available sickness absence data)	657 314 169	96 46 25	365 144 156	149–365 82–240 87–269

^a^ Participants who were still sick-listed after 12-month follow-up (end of studies)

^b^ The median is 365 days because < 50% returns to work during 12-month follow-up

### Missing values

Most variables did not have any missing values. Two variables (educational level and expected RTW < 6 months) were missing for > 50% of the participants because they were not available for one complete study. Therefore these variables were not included in the analyses. Sickness absence data were missing for < 5% of the participants and therefore did not need further inspection. Only two variables had > 5% missing values: RTW self-efficacy (34%) and psychological symptoms (34%). These data were missing because the baseline questionnaire of one study [24] was not fully filled in by 72% of the participants of that study. Additionally, four participants did not fully fill in the questions regarding RTW-self-efficacy. For both RTW self-efficacy and psychological symptoms, participants with missing values differed significantly compared to those without missing values. Furthermore, Little’s MCAR test was significant, also meaning that it is unlikely that the missing values are MCAR. The logistic regression analyses (Nagelkerke R²: 0.73 for RTW self-efficacy and 0.74 for psychological symptoms) showed that the missing values can be predicted by the other variables, and thus considered to be MAR. The missing values pattern did not show monotonicity, therefore multiple imputation was used. Imputation of 34 sets (because of 34% missing values, rule of thumb) was conducted for the Cox regression analyses.

### Censored data

The participants who did not return to work sustainably within 12 months (74%) or who were still on sick-leave after 12-month follow-up (50%) were considered to be right-censored in the analyses. They received a value of 365 days for both dependent variables. In a sensitivity analysis, all participants with 365 days as value for time until RTW or duration of sickness absence were changed into 0 days. This did not show different results in the Cox regression analyses.

### Proportional hazards assumption

The log-minus-log plot, scatter plots and regression analysis including time-dependency showed slight indications for non-proportionality in one independent variable (study allocation) in the analysis regarding time until RTW. However, the HR of the time-dependency was around 1 with a very small 95% CI (HR 1.002, 95% CI 1.000-1.004) and inclusion as time-dependent variable in the regression analysis did not show a different result. Therefore, this potential non-proportionality was considered meaningless.

### Cox regression: predictors for sustainable RTW

Results of the univariable and multivariable analyses regarding the potential predictors for a longer time until sustainable RTW are presented in Table 4. The univariable analyses showed three potential predictors (*p* < 0.2): age, baseline employment status and severity of psychological symptoms. The unadjusted survival curves are presented in Additional file 2. The VIF of the remaining predictors was < 10, meaning that there was no evidence for multicolllinearity. Therefore, the remaining potential predictors could be included in the multivariable analysis.

In the multivariable analysis age ≥ 50 years (HR 0.57, 95% CI 0.39–0.82), unemployment at baseline (HR 0.19, 95% CI 0.11–0.33), loss of employment contract during sickness absence (HR 0.25, 95% CI 0.14–0.47) and severe psychological symptoms (HR 0.64, 95% CI 0.43–0.93) were predictive (*p* < 0.05) for a longer time until sustainable RTW. Unemployment at baseline was the strongest predictor: the HR 0.19 (95% CI 0.11–0.33, *p* < 0.001) indicates that at any time point during 12-months follow-up 1/5 as many unemployed participants returned to work, compared to participants with temporary agency work. Consequently, the probability (PI) of a longer time until RTW for unemployed participants compared to participants with temporary agency work was 84%. The time until ≥ 25% of the participants with temporary agency work had a RTW was 98 days, compared to 249 days for the participants who lost their employment contract during sickness absence. The time until RTW could not be calculated for unemployed participants, because their RTW rate was < 25%. See Table 4 for the HRs and PIs, and Additional file 4 for time until 25% RTW for each predictor.

**Table 4:** Predictors of a longer time until sustainable RTW during 12-month follow-up (results of univariable and multivariable Cox regression, N = 681)

Independent variables	Univariable Cox regression			Multivariable Cox-regression				
	HR^a^	95% CI	*p*-value	HR	95% CI	*p*-value	PI^b^	95% CI
*Age*								
<50 yrs	1(ref)							
≥50 yrs	0.56	0.39–0.80	0.001*	0.57	0.39–0.82	0.002*	0.64	0.55–0.72
*Gender*								
Male	1(ref)							
Female	0.9	0.67–1.21	0.5					
*Employment status*								
Temporary agency	1(ref)							
Loss of employment contract^c^ Unemployed	0.31 0.19	0.17–0.57 0.11–0.33	< 0.001* < 0.001*	0.25 0.19	0.14–0.47 0.11–0.33	< 0.001* < 0.001*	0.80 0.84	0.68–0.88 0.75–0.90
*Psychological symptoms* ^d^								
Mild-to-moderate	1(ref)							
Severe	0.66	0.45–0.96	0.03*	0.64	0.43–0.93	0.02*	0.61	0.52–0.70
*RTW self-efficacy* ^e^								
High	1(ref)							
Low	0.94	0.63–1.39	0.8					
*Study*								
Brainwork	1(ref)							
Co-Work Cohort	0.95 0.88	0.67–1.35 0.61–1.27	0.8 0.5					
*Study allocation*								
Intervention	1(ref)							
Control Cohort	0.90 0.85	0.64–1.26 0.58–1.24	0.5 0.4					

^a^ An HR of < 1 indicates a longer time until RTW compared to the reference group

^b^ The PI indicates the probability of a longer time until RTW compared to the reference group. A PI > 0.5 indicates a higher probability

^c^ Loss of employment contract during sickness absence period

^d^ Cut-off point based on the median of the GHQ12 (Brainwork participants) and 4DSQ (Cohort and Co-Work participants)

^e^ Cut-off point based on the median of the RTW-SE scale (Brainwork participants) and ASE-SE subscale (Cohort and Co-Work participants)

*p < 0.05

### Cox regression: predictors for a prolonged duration of sickness absence

The results of univariable and multivariable Cox regression analyses about potential predictors for a longer time until end of sickness absence (= duration of sickness absence) are presented in Table 5. The univariable (unadjusted) survival curves are presented in Additional file 3. Based on the univariable analyses, four potential predictors (*p* < 0.2) were included in the multivariable analyses: gender, baseline employment status, severity of psychological symptoms and RTW self-efficacy. The VIF of these remaining predictors was < 10. Therefore, the remaining potential predictors could be included in the multivariable analysis. In the multivariable analyses male gender (HR 0.77, 95% CI 0.62–0.97), unemployment at baseline (HR 0.47, 95% CI 0.27–0.84), loss of employment contract during sickness absence (HR 0.48, 95% CI 0.26–0.90) and severe psychological symptoms (HR 0.64, 95% CI 0.46–0.87) were predictive for a prolonged duration of sickness absence (*p* < 0.05). RTW self-efficacy did emerge as potential predictor in the univariable analysis (HR 0.80, 95% CI 0.60–1.07, *p* = 0.1), but did not act as a predictor in the multivariable analysis (HR 0.86, 95% CI 0.63–1.17, *p* = 0.3). Baseline unemployment, loss of employment contract and severe symptoms were the strongest predictors for a longer duration of sickness absence. The probability of a prolonged duration of sickness absence in unemployed participants or participants who lost their employment contract was 68% compared to participants with temporary agency work. The time until ≥ 25% of the participants were no longer sick-listed was 180 days for participants who lost their employment contract, 145 days for unemployed participants and 58 days for participants with temporary agency work. See Table 5 for the HRs and PIs, and Additional file 4 for time until 25% end of sickness absence for each predictor.

**Table 5:** Predictors of a prolonged duration of sickness absence during 12-month follow-up (results of univariable and multivariable Cox regression, N = 657**)**

Independent variables	Univariable Cox regression			Multivariable Cox regression				
	HR^a^	95% CI	*p*-value	HR	95% CI	*p*-value	PI^b^	95% CI
*Age*								
<50 yrs	1(ref)							
≥50 yrs	0.99	0.78–1.26	0.9					
*Gender*								
Female	1(ref)							
Male	0.79	0.64–0.99	0.04*	0.77	0.62–0.97	0.03*	0.56	0.51–0.62
*Employment status*								
Temporary agency	1(ref)							
Loss of employment contract^c^ Unemployed	0.56 0.52	0.31–1.02 0.30–0.91	0.06 0.02*	0.48 0.47	0.26–0.90 0.27–0.84	0.02* 0.01*	0.68 0.68	0.53–0.79 0.54–0.79
*Psychological symptoms* ^d^								
Mild-to-moderate	1(ref)							
Severe	0.63	0.46–0.85	0.003*	0.64	0.46–0.87	0.005*	0.61	0.53–0.68
*RTW self-efficacy* ^e^								
High	1							
Low	0.80	0.60–1.07	0.1	0.86	0.63–1.17	0.3	0.54	0.46–0.61
*Study*								
Co-Work	1 (ref)							
Brainwork Cohort	0.95 0.96	0.73–1.23 0.71–1.31	0.7 0.8					
*Study allocation*								
Intervention	1 (ref)							
Control Cohort	0.87 0.93	0.68–1.12 0.70–1.24	0.3 0.6					

^a^ An HR of < 1 indicates a prolonged duration of sickness absence compared to the reference group

^b^ The PI indicates the probability of a prolonged duration of sickness absence compared to the reference group. A PI > 0.5 indicates a higher probability

^c^ Loss of employment contract during sickness absence period

^d^ Cut-off point based on the medians of the GHQ12 (Brainwork participants) and 4DSQ (Cohort and Co-Work participants)

^e^ Cut-off point based on the medians of the RTW-SE scale (Brainwork participants) and ASE-SE subscale (Cohort and Co-Work participants)

*p < 0.05

## Discussion

### General findings

This secondary analysis of three longitudinal studies aimed to identify predictors of a longer time until sustainable RTW and prolonged duration of sickness absence among sick-listed precarious workers with a CMD. The results show that severe psychological symptoms, unemployment at the moment of sick-listing and loss of employment contract during sickness absence were predictive of both a longer time until sustainable RTW and a prolonged duration of sickness absence. In addition, age above 50 years predicted a longer time until sustainable RTW and male gender a prolonged duration of sickness absence. Remarkably, a low level of RTW self-efficacy did emerge as potential predictor in the univariate analysis regarding a prolonged duration of sickness absence, but not in the multivariable analysis.

### Comparison with literature

The predictors that emerged from this secondary analysis are mainly in line with previous studies among sick-listed workers with a CMD. Unemployment and a lower level of socioeconomic status have also been found predictive of RTW in other studies [17, 20, 46, 47]. Severity of psychological symptoms has been associated with both RTW and duration of sickness absence in previous research [18, 20, 48]. Moreover, workers with more severe diagnoses such as depressive disorder are less likely to return to work compared to those with milder stress-related disorders [49–51]. Age is also a known predictor of RTW [18, 19, 49, 52] and of recurrent or long term disability pensions [17, 49]. Regarding the predictive role of gender, previous studies show conflicting results [17–19, 47]. An explanation of these inconsistent findings could be gender differences in health characteristics [53] and differences in home-related demands [54].

Only a few published studies specifically report on predictors of RTW or sickness absence among sick-listed precarious workers with CMD. A previous Dutch cohort study among precarious workers with mental disorders also found age, employment status and perceived health to be predictive of RTW [19]. Another study among Dutch sick-listed precarious workers showed different predictors regarding long-term sickness absence (educational level, expected duration of sickness absence and help-seeking behavior) [21]. However, this study was conducted among precarious workers with all types of mental or physical health complaints, and these potential predictors were not included in the current analyses. The predictors of RTW in the present study are also in line with the results of a large Finnish cohort study, including unemployed workers with a work history on full temporary disability benefits [47]. However, enrolment on a vocational rehabilitation program had a strong association with RTW among workers with mental disorders, while in our study allocation to a RTW intervention was not predictive. This could be a result of the low percentage of participants that actually received the full intervention in the included studies [23, 24]. Though, the per-protocol analysis of one of these studies [23] did not indicate different results. Another explanation could be that the participants of the included studies [23, 24] were randomly allocated to an intervention, while Laaksonen et al. [47] only evaluated a cohort without intervening. Therefore, participants of this Finnish study who received an intervention may have been selected based on their probabilities for successful RTW [47]. Also, the RTW interventions included in the current study [23, 24] differed from each other and also differed from the interventions of the Finnish study [47].

Remarkably, the level of RTW self-efficacy did not show associations with RTW and sickness absence in the present study. This deviates from several other studies, where a higher level of RTW self-efficacy has been found related to RTW and earlier RTW [19, 51, 52]. RTW self-efficacy is closely related to RTW expectations. Negative RTW expectations are also known to be predictive of RTW [18, 20, 51]. Neither did the current study show a difference between the participants in the cohort study (with lower levels of RTW-expectations) compared to the participants of the RCTs. Hypothetically, RTW self-efficacy could act differently for precarious workers, because of the lack of a job to return to, the influence of the social security system and fear of losing their benefit if returning to work [55]. The study of Lovvik et al. also showed a different predictive value of RTW-expectations in workers on long-term disability benefits compared to employed workers at risk of or on sick leave [56]. Furthermore, in the present study, only baseline RTW self-efficacy has been included, while change in RTW self-efficacy has been found predictive of RTW [57], and this change of RTW self-efficacy differs among individual workers with CMD [58]. Another explanation could be that the RTW self-efficacy questionnaires used in the included studies were not fully geared to measure the RTW self-efficacy in precarious workers, as these questionnaires were developed for workers with permanent work. An instrument for precarious workers has not yet been developed and tested.

### Strengths and limitations

As far as our knowledge is concerned, this is the first study analyzing data of several/multiple studies about predictors of RTW and sickness absence in this specific group of sick-listed workers. A strength of this study is the primary focus on precarious workers with CMD. Most previous studies only included employed workers with CMD. However, the Dutch social security system differs from other countries and, therefore, the results of this study may be less applicable to precarious workers in other countries. Another strength of this study is the use of registered data from the UWV database for the dependent variables and some independent variables. This meant there were almost no missing data for these outcomes. Moreover, these registered data are automatically collected through governmental computerized systems and therefore are more accurate than self-reported data [59, 60].

A limitation of this study is the presence of censored data. Most participants did not yet return to work during 12 months. A longer follow-up period of the included studies could have given more time points of RTW, which could have resulted in different predictive values of the independent variables. Another limitation is the use of data of different instruments regarding RTW-efficacy and severity of psychological symptoms. The use of the same instruments could have produced other results. Though most of these instruments are extensively validated and we only used the sum scores and corresponding medians to differentiate between high and low values, and not the individual items. Furthermore, not all potential predictors could be included in the analyses. Therefore the results are not a complete overview of predictors of RTW and duration of sickness absence among unemployed workers with CMD. Also, a meta-analysis with al larger number of included studies and participants could show different results. Finally, there is always the possibility that including potential predictors as time-varying covariates at the imputation stage could have led to (slightly) different results, because ignoring potential time-varying effects at the imputation stage could results in biased estimates according to recent statistical insights [61]. However, we extensively investigated the proportional hazards assumption using several statistical methods and we did not find enough evidence for non-proportionality.

### Implications for practice and research

The predictors found in this study can help occupational health and mental health professionals to recognize those sick-listed precarious workers with CMD at risk of long-term sickness absence and difficulties with sustainable RTW, enabling targeted interventions to be arranged to lower barriers to RTW and facilitate functional recovery. Unemployment and severe psychological symptoms are predictors of both a longer time until sustainable RTW and prolonged duration of sickness absence, making them potential barriers to actual RTW. For unemployed workers, specialised vocational rehabilitation support, such as supported employment [62], may be needed to help them find a new suitable job. Particularly in the event of long-term unemployment and age > 50 years, occupational rehabilitation may also address the skills needed regarding the rapid changes in the labor market (flexibilization, automation) to enhance job readiness and sustainable return to new work [63]. For workers with severe psychological symptoms, additional medical or psychological interventions focused on symptom reduction and improving functional recovery may be necessary. These interventions could be regular mental health interventions, but also (or subsequently) specific work-directed psychological interventions such as work-focused cognitive behavioral therapy [64]. For workers with both severe psychological symptoms and long-term unemployment a combination of psychological and occupational rehabilitation interventions could be beneficial.

However, these implications for occupational rehabilitation of precarious workers should be taken with caution, because there is no international consensus about the definition of precarious employment [14]. Also, all included studies were conducted in the Netherlands, a high-income country with a specific social security system. As a result, the type of socioeconomic and health-related vulnerability of Dutch precarious workers might differ compared to precarious workers in other countries [11].

More research is needed about predictors of RTW among other types of precarious workers with CMD. Furthermore, based on the contradictory results of the predictive value of RTW self-efficacy, more knowledge is needed about the role of RTW self-efficacy and about the effect of changes in RTW self-efficacy on RTW among precarious workers with CMD. Regarding gender differences, evaluation of the origin of these differences and the need for gender specific approaches in occupational rehabilitation for precarious workers with CMD is required. Also, there is a need for the development and evaluation of occupational rehabilitation interventions focusing on the presence of specific barriers to RTW for precarious workers with CMD. Based on the results of the current study, these interventions should focus on both a decrease of psychological symptoms and specific occupational support regarding long-term unemployment. A recent systematic review showed that a combination of work-directed interventions and mental health interventions leads to reduction of sickness absence days among employed workers with depressive disorder [65]. This also supports the need for scientific evaluation of combined interventions among precarious workers with CMD. However, previously evaluated interventions among Dutch precarious workers with CMD were not found effective, possibly due to the low level of protocol adherence [22, 23]. Therefore, it is important to focus on implementation of interventions during future studies too.

## Conclusions

Unemployment at the moment of sick-listing, loss of employment contract during sickness absence and severe psychological symptoms are predictive of both a longer time until sustainable RTW and prolonged duration of sickness absence in sick-listed precarious workers with CMD. Age above 50 years also predicts a longer time until sustainable RTW and male gender a prolonged duration of sickness absence. Knowledge about these predictors can assist occupational health and mental health professionals in the early recognition of those workers at risk of long-term sickness absence and long-term unemployment. Then, targeted occupational rehabilitation interventions and mental health interventions can be arranged to support functional recovery and return to (generally new) work. As far as our knowledge is concerned, this is the first study analyzing data of multiple/several trials regarding predictors of RTW and sickness absence in this specific group of sicklisted workers. More (pooled) research is needed about predictors, barriers and facilitors for RTW in sick-listed precarious workers with CMD, especially regarding the role and assessment of RTW self-efficacy.

### Electronic supplementary material

Below is the link to the electronic supplementary material.

Additional file 1: Table 1: baseline psychological symptoms and RTW-self-efficacy

Additional file 2: Figures 1, 2, 3, 4, 5, 6 and 7: Univariable, unadjusted survival curves illustrating time until sustainable return to work (RTW) stratified by baseline age, gender, employment status, psychological symptoms, RTW self-efficacy, study and study allocation

Additional file 3: Figures 8, 9, 10, 11, 12 and 13: Univariable, unadjusted survival curves illustrating time until end of sick leave (= duration of sickness absence) stratified by baseline age, gender, employment status, psychological symptoms, return to work (RTW) self-efficacy, study and study allocation

Additional file 4: Table 2, and 3: Median and 25th percentile time until sustainable return to work (RTW) and time until end of sick leave (= duration of sickness absence)

## Data Availability

The datasets generated during and/or analyzed during the current study are available from the corresponding author on reasonable request.

## List of Abbreviations

ASE-model: Attitude-Social influence-Self-Efficacy model
95% CI: 95% confidence interval
CMD: Common mental disorder
4DSQ: Four-Dimensional Symptom Questionnaire
GHQ-12: General Health Questionnaire-12
HR: Hazard ratio
IQR: Interquartile range
MAR: Missing ad random
MCAR: Missing completely ad random
MICE: Multiple imputation by chained equations
NMAR: Not missing at random
PI: Probabilistic index
RCT: Randomized controlled trial
RTW: Return to work
RTW-SE scale: Return To Work Self-Efficacy scale
SD: Standard deviation
TRIPOD: Transparent Reporting of a multivariable prediction model for Individual Prognosis or Diagnosis
UWV: Dutch Social Security Institute:the Institute for Employee Benefits Schemes (In Dutch:Uitvoeringsinstituut Werknemersverzekeringen)
VIF: Variance inflation factor

## References

[1] GBD 2019 Mental Disorders Collaborators (2022). Global, regional, and national burden of 12 mental disorders in 204 countries and territories, 1990–2019: a systematic analysis for the global burden of Disease Study 2019. The Lancet Psychiatry.

[2] Steel Z, Marnane C, Iranpour C, Chey T, Jackson JW, Patel V, Silove D (2014). The global prevalence of common mental disorders: a systematic review and meta-analysis 1980–2013. Int J Epidemiol.

[3] Ten Have M, Tuithof M, van Dorsselaer S, Schouten F, Luik AI, de Graaf R (2023). Prevalence and trends of common mental disorders from 2007-2009 to 2019‐2022: results from the Netherlands Mental Health Survey and Incidence studies (NEMESIS), including comparison of prevalence rates before vs. during the COVID‐19 pandemic. World Psychiatry.

[4] OECD (2021). Fitter minds, fitter jobs: from awareness to change in integrated mental health, skills and work policies.

[5] Saunders SL, Nedelec B (2014). What work means to people with work disability: a scoping review. J Occup Rehabil.

[6] Guhne U, Pabst A, Lobner M, Breilmann J, Hasan A, Falkai P, Kilian R, Allgower A, Ajayi K, Baumgartner J, Brieger P, Frasch K, Heres S, Jager M, Kuthmann A, Putzhammer A, Schneeweiss B, Schwarz M, Becker T, Kosters M, Riedel-Heller SG (2021). Employment status and desire for work in severe mental Illness: results from an observational, cross-sectional study. Soc Psychiatry Psychiatr Epidemiol.

[7] Modini M, Joyce S, Mykletun A, Christensen H, Bryant RA, Mitchell PB, Harvey SB (2016). The mental health benefits of employment: results of a systematic meta-review. Australas Psychiatry.

[8] Schuring M, Robroek SJ, Burdorf A (2017). The benefits of paid employment among persons with common mental health problems: evidence for the selection and causation mechanism. Scand J Work Environ Health.

[9] van der Noordt M, H IJ, Droomers M, Proper KI (2014). Health effects of employment: a systematic review of prospective studies. Occup Environ Med.

[10] Cygan-Rehm K, Kuehnle D, Oberfichtner M (2017). Bounding the causal effect of unemployment on mental health: nonparametric evidence from four countries. Health Econ.

[11] OECD (2018). The future of social protection: what works for non-standard workers?.

[12] Gutiérrez-Barbarrusa T (2016). The growth of precarious employment in Europe: concepts, indicators and the effects of the global economic crisis. Int Labour Rev.

[13] Kalleberg AL (2018). Job insecurity and well-being in rich democracies. Econ Soc Rev (Irel).

[14] Matilla-Santander N, Martin-Sanchez JC, Gonzalez-Marron A, Cartanya-Hueso A, Lidon-Moyano C, Martinez-Sanchez JM (2021). Precarious employment, unemployment and their association with health-related outcomes in 35 European countries: a cross-sectional study. Crit Public Health.

[15] Ronnblad T, Gronholm E, Jonsson J, Koranyi I, Orellana C, Kreshpaj B, Chen L, Stockfelt L, Bodin T (2019). Precarious employment and mental health: a systematic review and meta-analysis of longitudinal studies. Scand J Work Environ Health.

[16] Ervasti J, Vahtera J, Virtanen P, Pentti J, Oksanen T, Ahola K, Kivimaki M, Virtanen M (2014). Is temporary employment a risk factor for work disability due to depressive disorders and delayed return to work? The Finnish Public Sector Study. Scand J Work Environ Health.

[17] Cornelius LR, van der Klink JJ, Groothoff JW, Brouwer S (2011). Prognostic factors of long term disability due to mental disorders: a systematic review. J Occup Rehabil.

[18] De Vries H, Fishta A, Weikert B, Sanchez AR, Wegewitz U (2018). Determinants of sickness absence and return to work among employees with common mental disorders: a scoping review. J Occup Rehabil.

[19] Etuknwa A, Daniels K, Eib C (2019). Sustainable return to work: a systematic review focusing on personal and social factors. J Occup Rehabil.

[20] Audhoe SS, Hoving JL, Nieuwenhuijsen K, Friperson R, de Jong PR, Sluiter JK, Frings-Dresen MH (2012). Prognostic factors for the work participation of sick-listed unemployed and temporary agency workers with psychological problems. J Occup Rehabil.

[21] Louwerse I, van Rijssen HJ, Huysmans MA, van der Beek AJ, Anema JR (2020). Predicting long-term sickness absence and identifying subgroups among individuals without an employment contract. J Occup Rehabil.

[22] Ekberg K, Pransky GS, Besen E, Fassier JB, Feuerstein M, Munir F, Blanck P, Hopkinton Conference Working Group on Workplace Disability Prevention. &. New business structures creating organizational opportunities and challenges for work disability prevention. J Occup Rehabil. 2016;26(4):480-9. 10.1007/s10926-016-9671-0 PMC5104761 27704343

[23] Lammerts L, Schaafsma FG, Bonefaas-Groenewoud K, van Mechelen W, Anema J (2016). Effectiveness of a return-to-work program for workers without an employment contract, sick-listed due to common mental disorders. Scand J Work Environ Health.

[24] Audhoe SS, Hoving JL, Zijlstra BJH, Frings-Dresen MHW, Nieuwenhuijsen K (2021). Is the Brainwork Intervention effective in reducing sick leave for non-permanent workers with psychological problems? Results of a controlled clinical trial. BMC Public Health.

[25] Moons KG, Altman DG, Reitsma JB, Ioannidis JP, Macaskill P, Steyerberg EW, Vickers AJ, Ransohoff DF, Collins GS (2015). Transparent reporting of a multivariable prediction model for individual prognosis or diagnosis (TRIPOD): explanation and elaboration. Ann Intern Med.

[26] Young AE, Viikari-Juntura E, Boot CRL, Chan C, Ruiz de Porras D, Linton SJ (2016). Workplace outcomes in Work-Disability Prevention Research: a review with recommendations for Future Research. J Occup Rehabil.

[27] Sikora A, Schneide G, Stegmann R, Wegewitz U. (2019). Returning to work after sickness absence due to common mental disorders: study design and baseline findings from an 18 months mixed methods follow-up study in Germany. BMC Public Health. 2019;19(1): 1–13. 10.1186/s12889-019-7999-z PMC6902352 31823752

[28] Goldberg DP, Gater R, Sartorius N, Ustun TB, Piccinelli M, Gureje O, Rutter C (1997). The validity of two versions of the GHQ in the WHO study of mental Illness in general health care. Psychol Med.

[29] Terluin B, van Marwijk HW, Ader HJ, de Vet HC, Penninx BW, Hermens ML, van Boeijen CA, van Balkom AJ, van der Klink JJ, Stalman WA (2006). The four-Dimensional Symptom Questionnaire (4DSQ): a validation study of a multidimensional self-report questionnaire to assess distress, depression, anxiety and somatization. BMC Psychiatry.

[30] Gnambs T, Staufenbiel (2018). The structure of the General Health Questionnaire (GHQ-12): two meta-analytic factor analyses. Health Psychol Rev.

[31] Terluin B, Brouwers EP, van Marwijk HW, Verhaak PF, van der Horst HE (2009). Detecting depressive and anxiety disorders in distressed patients in primary care; comparative diagnostic accuracy of the four-Dimensional Symptom Questionnaire (4DSQ) and the hospital anxiety and Depression Scale (HADS). BMC Fam Pract.

[32] Terluin B, Oosterbaan DB, Brouwers EP, van Straten A, van de Ven PM, Langerak W, van Marwijk HW (2014). To what extent does the anxiety scale of the four-Dimensional Symptom Questionnaire (4DSQ) detect specific types of anxiety disorder in primary care? A psychometric study. BMC Psychiatry.

[33] Bandura A (1977). Self-efficacy: toward a unifying theory of behavioral change. Psychol Rev.

[34] Lagerveld SE, Blonk RW, Brenninkmeijer V, Schaufeli WB (2010). Return to work among employees with mental health problems: development and validation of a self-efficacy questionnaire. Work Stress.

[35] Gjengedal RG, Lagerveld SE, Reme SE, Osnes K, Sandin K, Hjemdal O (2021). The return-to-work self-efficacy questionnaire (RTW-SE): a validation study of predictive abilities and cut-off values for patients on sick leave due to anxiety or depression. J Occup Rehabil.

[36] Van Oostrom SH, van Mechelen W, Terluin B, de Vet HC, Knol DL, Anema JR (2010). A workplace intervention for sick-listed employees with distress: results of a randomised controlled trial. Occup Environ Med.

[37] De Vries H, Dijkstra M, Kuhlman P (1988). Self-efficacy: the third factor besides attitude and subjective norm as a predictor of behavioural intentions. Health Educ Res.

[38] De Neve J, Gerds TA (2020). On the interpretation of the hazard ratio in Cox regression. Biometrical J.

[39] O’Brien RM (2007). A caution regarding rules of thumb for variance inflation factors. Qual Quant.

[40] Rubin DB (1976). Inference and missing data. Biometrika.

[41] Van Buuren S. Flexible imputation of missing data: 2nd edition. Boca Raton: CRC Press, Taylor & Francis Group; 2018.

[42] Little RJA (1988). A test of missing completely at random for multivariate data with missing values. J Am Stat Assoc.

[43] Nagelkerke NJD (1991). A note on a general definition of the coefficient of determination. Biometrika.

[44] White IR, Royston P, Wood AM (2011). Multiple imputation using chained equations: issues and guidance for practice. Stat Med.

[45] Wood AM, White IR, Royston P (2008). How should variable selection be performed with multiply imputed data?. Stat Med.

[46] Lammerts L, Schaafsma FG, Eikelenboom M, Vermeulen SJ, van Mechelen W, Anema JR, Penninx BW (2016). Longitudinal associations between biopsychosocial factors and sustainable return to work of sick-listed workers with a depressive or anxiety disorder. J Occup Rehabil.

[47] Laaksonen M, Gould R (2015). Return to work after temporary disability pension in Finland. J Occup Rehabil.

[48] Lagerveld SE, Bultmann U, Franche RL, van Dijk FJ, Vlasveld MC, van der Feltz-Cornelis CM, Bruinvels DJ, Huijs JJ, Blonk RW, van der Klink JJ, Nieuwenhuijsen K (2010). Factors associated with work participation and work functioning in depressed workers: a systematic review. J Occup Rehabil.

[49] Mattila-Holappa P, Ervasti J, Joensuu M, Ahola K, Pentti J, Oksanen T, Vahtera J, Kivimaki M, Virtanen M (2017). Do predictors of return to work and recurrence of work disability due to mental disorders vary by age? A cohort study. Scand J Public Healt.

[50] Sakakibara S, Sado M, Ninomiya A, Arai M, Takahashi S, Ishihara C, Miura Y, Tabuchi H, Shirahase J, Mimura M (2019). Predictive factors of the duration of sick leave due to mental disorders. Int J Ment Health Syst.

[51] Sikora A, Schneider G, Wegewitz U, Bultmann U (2022). Employees receiving inpatient treatment for common mental disorders in Germany: factors associated with time to first and full return to work. J Occup Rehabil.

[52] Nigatu YT, Liu Y, Uppal M, McKinney S, Gillis K, Rao S, Wang J (2017). Prognostic factors for return to work of employees with common mental disorders: a meta-analysis of cohort studies. Soc Psychiatry Psychiatr Epidemiol.

[53] De Rijk A, Janssen N, Alexanderson K, Nijhuis F (2008). Gender differences in return to work patterns among sickness absentees and their associations with health: a prospective cohort study in the Netherlands. Int J Rehabil Res.

[54] Nybergh L, Bergstrom G, Hellman T (2020). Do work- and home-related demands and resources differ between women and men during return-to-work? A focus group study among employees with common mental disorders. BMC Public Health.

[55] Suijkerbuijk Y, Nieuwenhuijsen K. Identification of the return-to-work mode in unemployed workers with mental health issues: A focus group study among occupational health professionals. Work. 2022 (pre-press): 1–16. 10.3233/WOR-210434 35527604

[56] Lovvik C, Shaw W, Overland S, Reme SE (2014). Expectations and Illness perceptions as predictors of benefit recipiency among workers with common mental disorders: secondary analysis from a randomised controlled trial. Bmj Open.

[57] Lagerveld SE, Brenninkmeijer V, Blonk RW, Twisk J, Schaufeli WB (2017). Predictive value of work-related self-efficacy change on RTW for employees with common mental disorders. Occup Environ Med.

[58] Horn L, Spronken M, Brouwers EPM, de Reuver RSM, Joosen MCW (2022). Identifying return to work self-efficacy trajectories in employees with mental health problems. J Occup Rehabil.

[59] Pole JD, Franche RL, Hogg-Johnson S, Vidmar M, Krause N (2006). Duration of work disability: a comparison of self-report and administrative data. Am J Ind Med.

[60] Johns G, Miraglia M (2015). The reliability, validity, and Accuracy of Self-reported absenteeism from work: a Meta-analysis. J Occup Health Psych.

[61] Keogh RH, Morris TP (2018). Multiple imputation in Cox regression when there are time-varying effects of covariates. Stat Med.

[62] Bejerholm U, Larsson ME, Johanson S (2017). Supported employment adapted for people with affective disorders-A randomized controlled trial. J Affect Disord.

[63] OECD (2018). Good jobs for all in a changing world of work: the OECD jobs strategy.

[64] Reme SE, Grasdal AL, Lovvik C, Lie SA, Overland S (2015). Work-focused cognitive-behavioural therapy and individual job support to increase work participation in common mental disorders: a randomised controlled multicentre trial. Occup Environ Med.

[65] Nieuwenhuijsen K, Verbeek JH, Neumeyer-Gromen A, Verhoeven AC, Bultmann U, Faber B (2020). Interventions to improve return to work in depressed people. Cochrane Database Syst Rev.

