# Supplementary material for: Predictors of time until return to work and duration of sickness absence in sick-listed precarious workers with common mental disorders: a secondary data-analysis of two trials and one cohort study
Source: Int J Ment Health Syst. 2023 Dec 8;17:48. doi: 10.1186/s13033-023-00613-7 (PMC10704639; doi:10.1186/s13033-023-00613-7)
Supplement: Supplementary file 1 — Additional file 1: Table 1: baseline psychological symptoms and RTW-self-efficacy [file 13033_2023_613_MOESM1_ESM.docx]

**Supplementary Information: Additional file 1**

*Title*: Predictors of time until return to work and duration of sickness absence in sick-listed precarious workers with common mental disorders: a secondary data-analysis of two trials and one cohort study.

*Authors*: Yvonne Suijkerbuijk, Frederieke Schaafsma, Lyanne Jansen, Selwin Audhoe, Lieke Lammers, Johannes Anema, Karen Nieuwenhuijsen

*Corresponding author*: Yvonne Suijkerbuijk, Amsterdam UMC, location University of Amsterdam, Department of Public and Occupational Health, Meibergdreef 9, 1105 AZ Amsterdam, The Netherlands. E: y.b.suijkerbuijk@amsterdamumc.nl

**Supplementary Table 1.** Baseline psychological symptoms and RTW-self-efficacy

|  | **Brainwork**  **N = 89)** | **Co-WORK**  **(N = 186)** | **Cohort**  **(N = 175)** |
| --- | --- | --- | --- |
| *Psychological symptoms*  GHQ-12ᵃ  4DSQᵇ anxiety  4DSQ depression  4DSQ distress  4DSQ somatization | 31.00 (25.00-34.00)  -  -  -  - | 10.00 (5.00-15.00)  7.00 (3.00-10.00)  27.00 (23.00-31.00)  14.00 (10.92-20.00) | 10.00 (5.00-16.00)  8.00 (4.00-12.00)  28.00 (25/00-31.00)  17.00 (12.00-23.00) |
| *RTW Self-efficacy*  RTW-SEᶜ  ASE-SEᵉ | 2.73 (1.95-3.55)ᵈ  - | -  6.00 (5.00-7.00) | -  8.00 (7.00-10.00) |

ᵃGHQ-12: General Health Questionnaire-12

ᵇ4DSQ: Four-Dimensional Symptom Questionnaire

ᶜRTW-SE: Return To Work Self-Efficacy scale

ᵈn = 85

**ᵉ**ASE-SE: Attitude-Social influence-Self-Efficacy questionnaire, Self-Efficacy subscale
