# Supplementary material for: Predictors of time until return to work and duration of sickness absence in sick-listed precarious workers with common mental disorders: a secondary data-analysis of two trials and one cohort study
Source: Int J Ment Health Syst. 2023 Dec 8;17:48. doi: 10.1186/s13033-023-00613-7 (PMC10704639; doi:10.1186/s13033-023-00613-7)

**Supplementary Information: Additional file 2**

*Title*: Predictors of time until return to work and duration of sickness absence in sick-listed precarious workers with common mental disorders: a secondary data-analysis of two trials and one cohort study.

*Authors*: Yvonne Suijkerbuijk, Frederieke Schaafsma, Lyanne Jansen, Selwin Audhoe, Lieke Lammers, Johannes Anema, Karen Nieuwenhuijsen

*Corresponding author*: Yvonne Suijkerbuijk, Amsterdam UMC, location University of Amsterdam, Department of Public and Occupational Health, Meibergdreef 9, 1105 AZ Amsterdam, The Netherlands. E: [y.b.suijkerbuijk@amsterdamumc.nl](mailto:y.b.suijkerbuijk@amsterdamumc.nl)

**Supplementary Figures 1-7: Univariable, unadjusted survival curves illustrating time until sustainable return to work (RTW) stratified by baseline *age, gender, employment status, psychological symptoms, RTW self-efficacy, study and study allocation.***

**Figure 1. Unadjusted survival curve illustrating time until sustainable RTW stratified by *age***


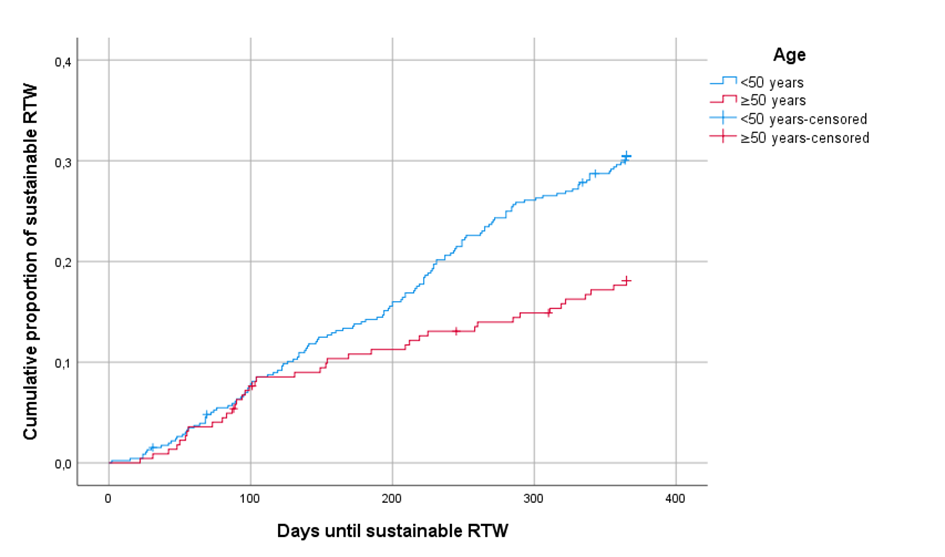


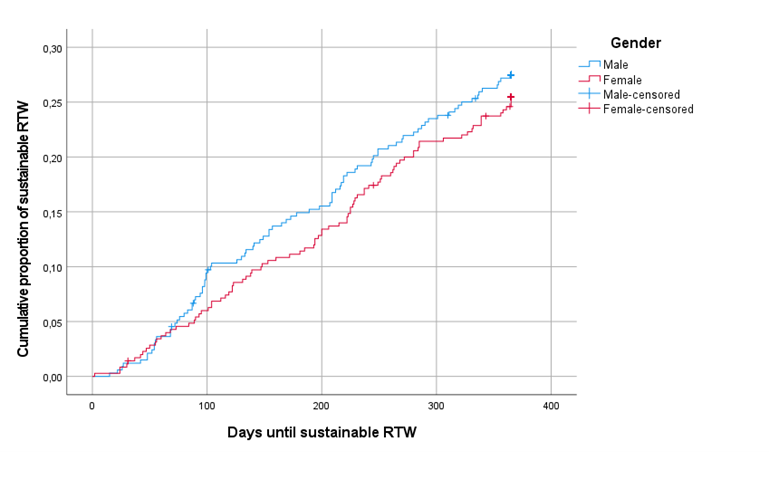
**Figure 2. Unadjusted survival curve illustrating time until sustainable RTW stratified by *gender***


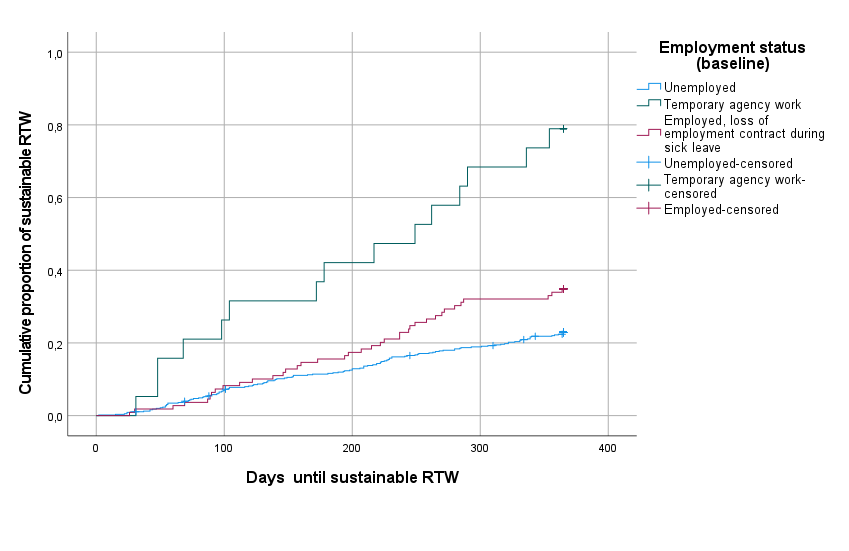
**Figure 3. Unadjusted survival curve illustrating time until sustainable RTW stratified by *employment status***

**Figure 4. Unadjusted survival curve illustrating time until sustainable RTW stratified by *psychological symptoms***


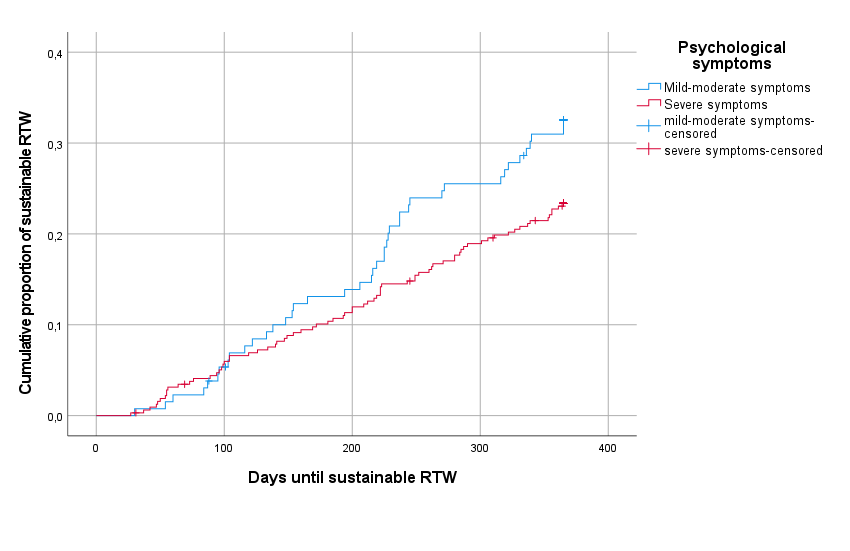


**Figure 5. Unadjusted survival curve illustrating time until sustainable RTW stratified by *RTW self-efficacy***


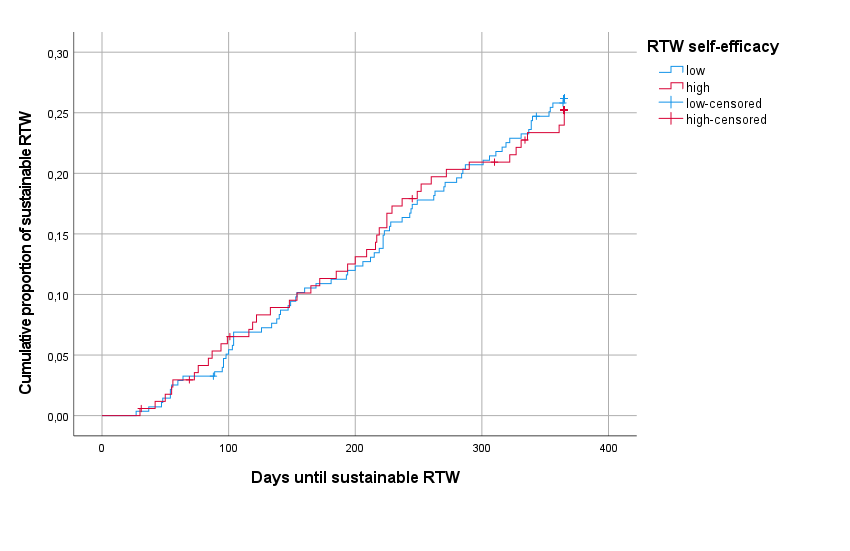


**Figure 6. Unadjusted survival curve illustrating time until sustainable RTW stratified by *study***


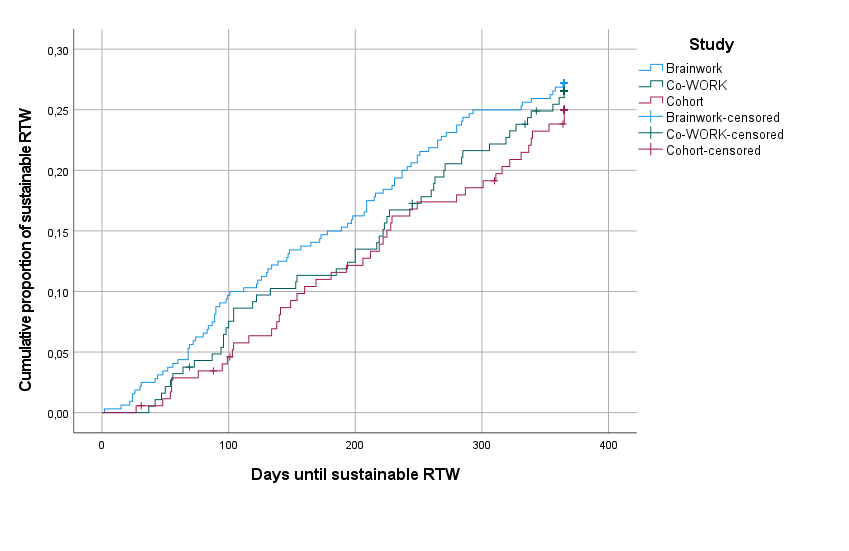


**Figure 7. Unadjusted survival curve illustrating time until sustainable RTW stratified by *study allocation***


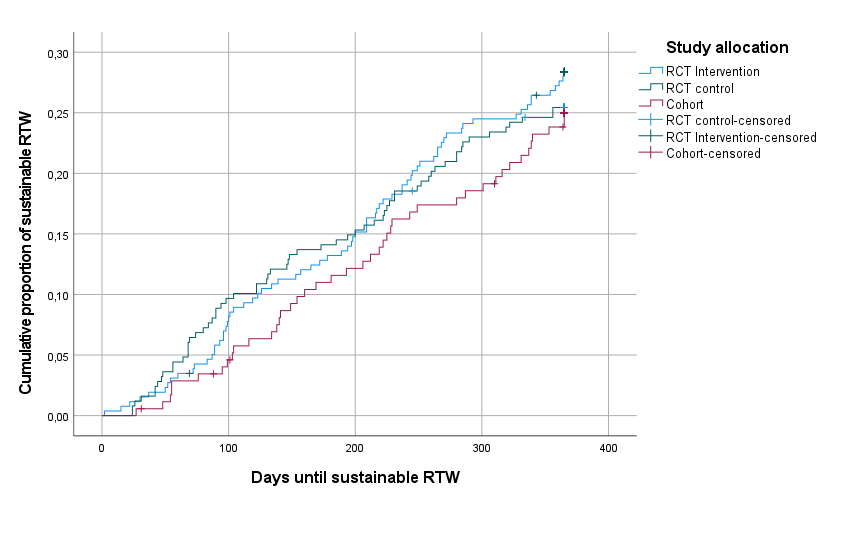

Supplement: Supplementary file 2 — Additional file 2: Figures 1, 2, 3, 4, 5, 6 and 7: Univariable, unadjusted survival curves illustrating time until sustainable return to work (RTW) stratified by baseline age, gender, employment status, psychological symptoms, RTW self-efficacy, study and study allocation [file 13033_2023_613_MOESM2_ESM.docx]
