# Supplementary material for: Predictors of time until return to work and duration of sickness absence in sick-listed precarious workers with common mental disorders: a secondary data-analysis of two trials and one cohort study
Source: Int J Ment Health Syst. 2023 Dec 8;17:48. doi: 10.1186/s13033-023-00613-7 (PMC10704639; doi:10.1186/s13033-023-00613-7)
Supplement: Supplementary file 3 — Additional file 3: Figures 8, 9, 10, 11, 12 and 13: Univariable, unadjusted survival curves illustrating time until end of sick leave (= duration of sickness absence) stratified by baseline age, gender, employment status, psychological symptoms, return to work (RTW) self-efficacy, study and study allocation [file 13033_2023_613_MOESM3_ESM.docx]

**Supplementary Information: Additional file 3**

*Title*: Predictors of time until return to work and duration of sickness absence in sick-listed precarious workers with common mental disorders: a secondary data-analysis of two trials and one cohort study.

*Authors*: Yvonne Suijkerbuijk, Frederieke Schaafsma, Lyanne Jansen, Selwin Audhoe, Lieke Lammers, Johannes Anema, Karen Nieuwenhuijsen

*Corresponding author*: Yvonne Suijkerbuijk, Amsterdam UMC, location University of Amsterdam, Department of Public and Occupational Health, Meibergdreef 9, 1105 AZ Amsterdam, The Netherlands. E: [y.b.suijkerbuijk@amsterdamumc.nl](mailto:y.b.suijkerbuijk@amsterdamumc.nl)

**Supplementary Figures 8-13: Univariable, unadjusted survival curves illustrating time until end of sick leave (=duration of sickness absence) stratified by baseline *age, gender, employment status, psychological symptoms, return to work (RTW) self-efficacy, study and study allocation.***

**Figure 8. Unadjusted survival curve illustrating time until end of sick leave stratified by *age***


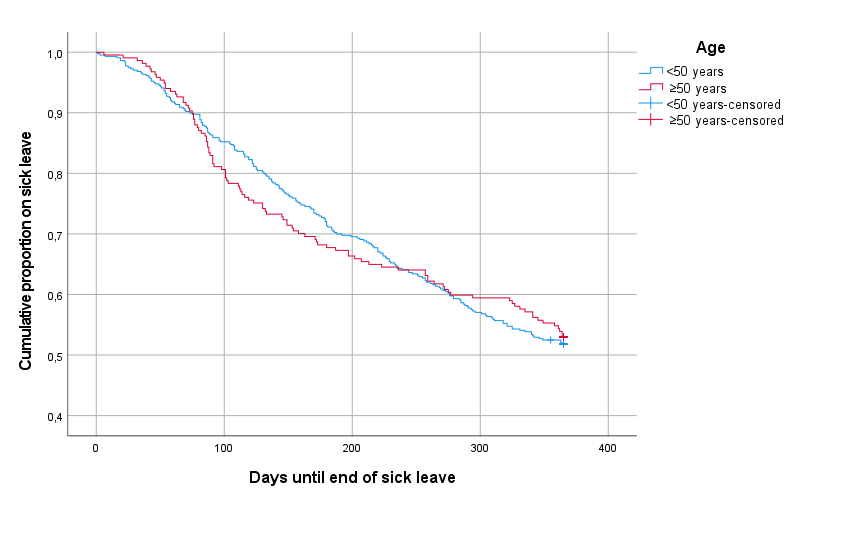


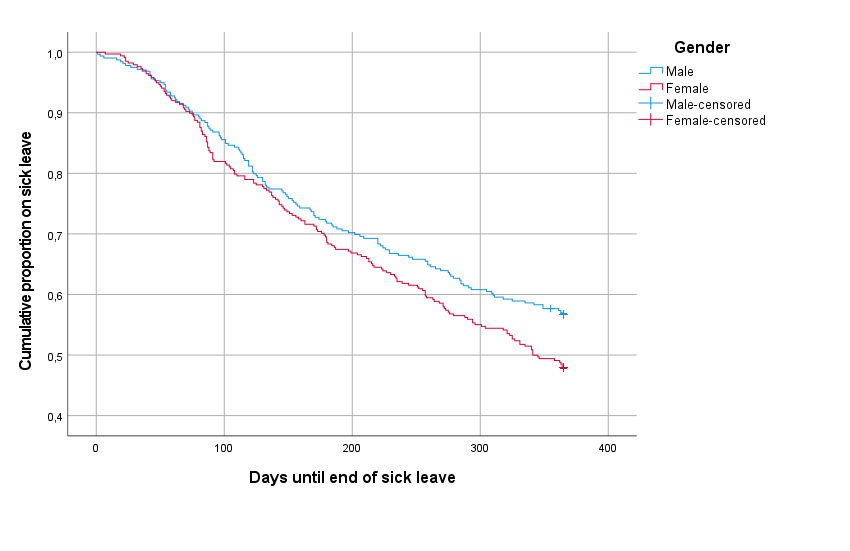
**Figure 9. Unadjusted survival curve illustrating time until end of sick leave stratified by *gender***


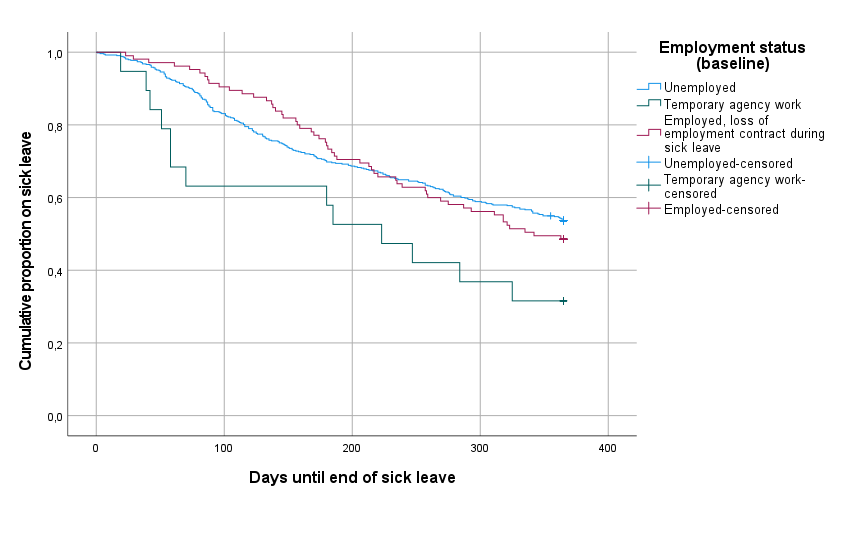
**Figure 10. Unadjusted survival curve illustrating time until end of sick leave stratified by *employment status***


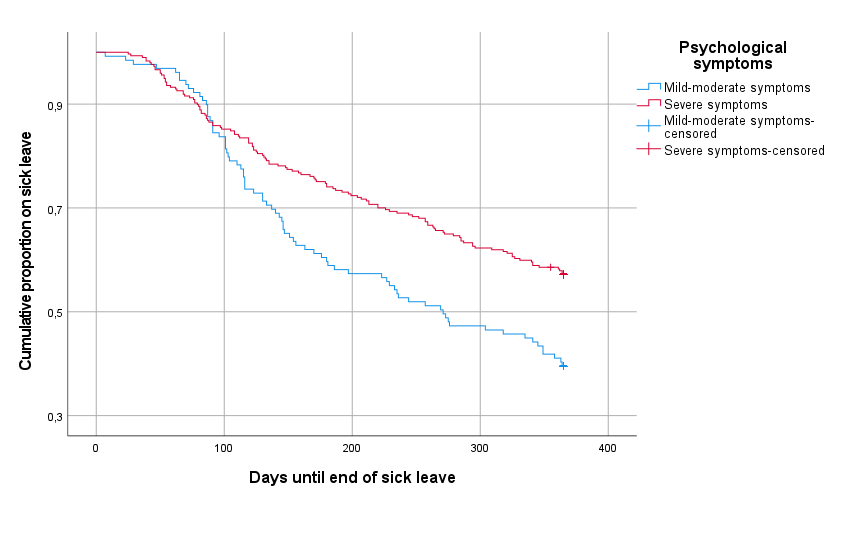
**Figure 11. Unadjusted survival curve illustrating time until end of sick leave stratified by *psychological symptoms***


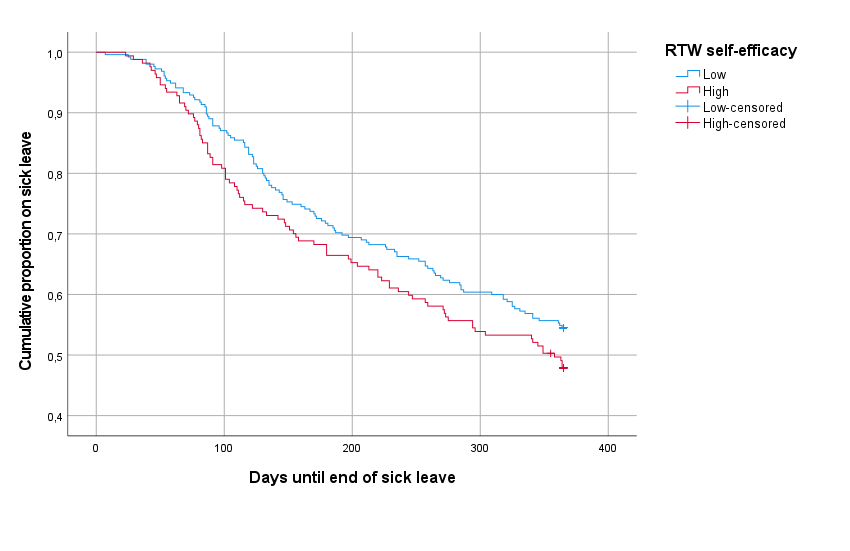
**Figure 12. Unadjusted survival curve illustrating time until end of sick leave stratified by *RTW self-efficacy***


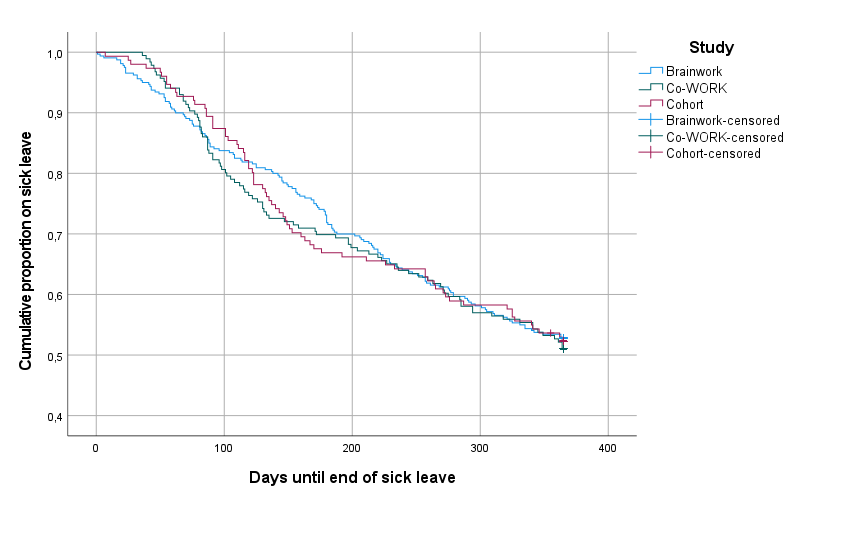
**Figure 13. Unadjusted survival curve illustrating time until end of sick leave stratified by *study***


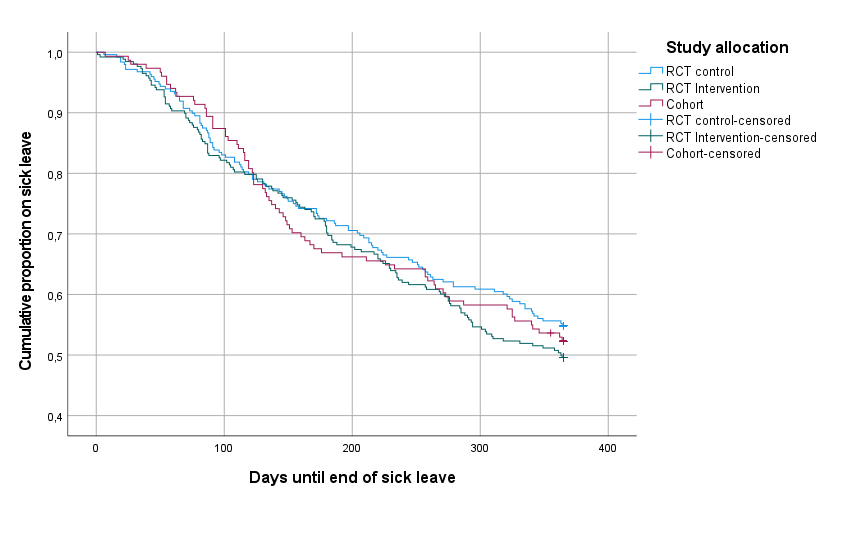
**Figure 14. Unadjusted survival curve illustrating time until end of sick leave stratified by *study allocation***
