# Supplementary material for: Predictors of time until return to work and duration of sickness absence in sick-listed precarious workers with common mental disorders: a secondary data-analysis of two trials and one cohort study
Source: Int J Ment Health Syst. 2023 Dec 8;17:48. doi: 10.1186/s13033-023-00613-7 (PMC10704639; doi:10.1186/s13033-023-00613-7)
Supplement: Supplementary file 4 — Additional file 4: Table 2, and 3: Median and 25th percentile time until sustainable return to work (RTW) and time until end of sick leave (= duration of sickness absence) [file 13033_2023_613_MOESM4_ESM.docx]

**Supplementary Information: Additional file 4**

*Title*: Predictors of time until return to work and duration of sickness absence in sick-listed precarious workers with common mental disorders: a secondary data-analysis of two trials and one cohort study.

*Authors*: Yvonne Suijkerbuijk, Frederieke Schaafsma, Lyanne Jansen, Selwin Audhoe, Lieke Lammers, Johannes Anema, Karen Nieuwenhuijsen

*Corresponding author*: Yvonne Suijkerbuijk, Amsterdam UMC, location University of Amsterdam, Department of Public and Occupational Health, Meibergdreef 9, 1105 AZ Amsterdam, The Netherlands. E: [y.b.suijkerbuijk@amsterdamumc.nl](mailto:y.b.suijkerbuijk@amsterdamumc.nl)

**Supplementary Tables 2-3:** Median and 25^th^ percentile time until sustainable return to work (RTW) and time until end of sick leave (=duration of sickness absence)

**Supplementary Table 2.** Median and 25^th^ percentile time until *sustainable RTW*

|  | **n (%) RTW** | **Days until ≥25% RTW (SEᵃ)** | **Days until ≥50% RTW (SE)** |
| --- | --- | --- | --- |
| *All participants* | 179 (26%) | 353 (-) |  |
| *Age*  <50 yrs  ≥50 yrs | 139 (30%)  40 (18%) | 284 (27)  - |  |
| *Gender*  Male  Female | 90 (27%)  89 (25%) | 322 (-)  365 (-) |  |
| *Employment status (baseline)*  Temporary agency  Loss of employment contract  Unemployed | 15 (79%)  38 (35%)  126 (23%) | 98 (36)  249 (25)  - | 249 (61) |
| *Psychological symptomsᵇ*  Mild-to-moderate  Severe | 42 (32%)  74 (23%) | 272 (40)  - |  |
| *RTW self-efficacyᶜ*  High  Low | 42 (25%)  72 (26%) | 365 (-)  353 (-) |  |
| *Study*  Brainwork  Co-Work  Cohort | 87 (37%)  49 (36%)  43 (35%) | 293 (-)  356 (-)  365 (-) |  |
| *Study allocation*  Intervention  Control  Cohort | 73 (28%)  63 (25%)  43 (25%) | 331 (-)  356 (-)  365 (-) |  |

ᵃSome standard errors (SE) could not be calculated, because the estimates of the survival distribution at percentile (25%

or 50%) +/- 5 were not available.

ᵇCut-off point based on the median of the GHQ12 (Brainwork participants) and 4DSQ (Cohort and Co-Work participants).

ᶜCut-off point based on the median of the RTW-SE scale (Brainwork participants) and ASE-SE subscale (Cohort and Co-Work participants).

**Supplementary Table 3.** Median and 25^th^ percentile time until *end of sick leave*

|  | **N (%) end of sickness absence** | **Days until ≥ 25% end of sickness leave (SEᵃ)** | **Days until ≥ 50% end of sick leave (SE)** |
| --- | --- | --- | --- |
| *All participants* | 314 (48%) | 149 (11) |  |
| *Age*  <50 yrs  ≥50 yrs | 212 (48%)  122 (47%) | 130 (12)  159 (17) |  |
| *Gender*  Male  Female | 138 (43%)  176 (52%) | 156 (19)  143 (16) | 341 (-) |
| *Employment status (baseline)*  Temporary agency  Loss of employment contract  Unemployed | 13 (68%)  54 (51%)  247 (46%) | 58 (11)  180 (20)  145 (12) | 223 (49)  342 (-) |
| *Psychological symptomsᵇ*  Mild-to-moderate  Severe | 78 (61%)  127 (43%) | 116 (13)  179 (23) | 271 (46) |
| *RTW self-efficacyᶜ*  High  Low | 87 (52%)  116 (45%) | 116 (17)  153 (17) | 358 (-) |
| *Study*  Brainwork  Co-Work  Cohort | 151 (47%)  91 (49%)  72 (48%) | 170 (12)  154 (22)  137 13) |  |
| *Study allocation*  Intervention  Control  Cohort | 130 (50%)  112 (45%)  72 (48%) | 157 (19)  154 (24)  137 (13) | 363 (-) |

ᵃSome standard errors (SE) could not be calculated, because the estimates of the survival distribution at percentile (25% or 50%) +/- 5 were not available.

ᵇCut-off point based on the median of the GHQ12 (Brainwork participants) and 4DSQ (Cohort and Co-Work participants).

ᶜCut-off point based on the median of the RTW-SE scale (Brainwork participants) and ASE-SE subscale (Cohort and Co-Work participants).
